# Fibroblast Diversity and Epigenetic Regulation in Cardiac Fibrosis

**DOI:** 10.3390/ijms25116004

**Published:** 2024-05-30

**Authors:** Laura Pilar Aguado-Alvaro, Nerea Garitano, Beatriz Pelacho

**Affiliations:** 1 Department of Biochemistry and Genetics, University of Navarra, 31008 Pamplona, Spain; laguado.3@unav.es (L.P.A.-A.); ngaritano@unav.es (N.G.); 2 Program of Cardiovascular Disease, Center for Applied Medical Research (CIMA), University of Navarra, 31008 Pamplona, Spain; 3 Instituto de Investigación Sanitaria de Navarra (IdiSNA), 31008 Pamplona, Spain

**Keywords:** cardiac fibroblast, transcriptomics, epigenetics, fibrosis, therapeutic approach

## Abstract

Cardiac fibrosis, a process characterized by excessive extracellular matrix (ECM) deposition, is a common pathological consequence of many cardiovascular diseases (CVDs) normally resulting in organ failure and death. Cardiac fibroblasts (CFs) play an essential role in deleterious cardiac remodeling and dysfunction. In response to injury, quiescent CFs become activated and adopt a collagen-secreting phenotype highly contributing to cardiac fibrosis. In recent years, studies have been focused on the exploration of molecular and cellular mechanisms implicated in the activation process of CFs, which allow the development of novel therapeutic approaches for the treatment of cardiac fibrosis. Transcriptomic analyses using single-cell RNA sequencing (RNA-seq) have helped to elucidate the high cellular diversity and complex intercellular communication networks that CFs establish in the mammalian heart. Furthermore, a significant body of work supports the critical role of epigenetic regulation on the expression of genes involved in the pathogenesis of cardiac fibrosis. The study of epigenetic mechanisms, including DNA methylation, histone modification, and chromatin remodeling, has provided more insights into CF activation and fibrotic processes. Targeting epigenetic regulators, especially DNA methyltransferases (DNMT), histone acetylases (HAT), or histone deacetylases (HDAC), has emerged as a promising approach for the development of novel anti-fibrotic therapies. This review focuses on recent transcriptomic advances regarding CF diversity and molecular and epigenetic mechanisms that modulate the activation process of CFs and their possible clinical applications for the treatment of cardiac fibrosis.

## 1. Introduction

Cardiovascular diseases (CVDs) are the leading cause of morbidity and mortality worldwide with an estimated value of 18 million deaths per year, which constitutes one-third of global total deaths [1]. Unfortunately, predictions are not favorable, and it is estimated that by 2030 the number increases to 24 million deaths per year, representing 42% of deaths worldwide [2]. Cardiac fibrosis is a common outcome of many CVDs, and its irreversibility and unstoppable progression constitute a real challenge to the scientific and medical communities. As occurs in other organ fibrotic diseases, cardiac fibrosis originates as the result of fibroblast activation, which once activated, begin to proliferate, differentiate into myofibroblasts, and secrete high amounts of extracellular matrix (ECM) proteins, hardening the cardiac muscle and impairing the original functionality of the heart. Although in some cases such as myocardial infarction (MI), fibrosis is initially beneficial for healing (avoiding ventricular wall rupture), its persistence and expansion afterwards joined to the negligible proliferative potential of adult cardiomyocytes, comprises the regeneration of the heart, and contributes, in the end, to heart failure progression and death. Nowadays, there are no effective therapies for the treatment of cardiac fibrosis [3], with heart transplantation being the only curative approach for advanced-stage patients, an extreme measure limited by the low availability of donors [4]. Despite constituting only 5–10% of the total heart cell content, cardiac fibroblasts (CFs) play an essential role in the development of myocardial fibrosis, as they coordinate chemical, mechanical, and electrical signals between the cellular and extracellular components of the heart [5]. In recent years, many studies have focused on a better understanding of the cellular and molecular mechanisms implicated in the CF activation process for the development of novel anti-fibrotic therapies that would help to decrease the vast morbidity and mortality linked to CVDs. In this review, we highlight the main findings obtained using single-cell transcriptomics and epigenetics in the knowledge of CF diversity and activation regulatory mechanisms, which might facilitate the emergence of targeted anti-fibrotic therapies.

## 2. Cardiac Fibroblasts/Myofibroblasts: Cellular and Molecular Characteristics

CFs are essential myocardial cells for maintaining a homeostatic ECM structure and normal heart function. However, stress and pathological conditions induce CF activation and differentiation into an ECM-producing cell state known as myofibroblast, which is the main active cell involved in the development of the adverse remodeling that triggers cardiac fibrosis. The study of CFs and myofibroblasts has been hampered due to the lack of sensitive and specific markers that identify these cell types. In the past, CFs were identified and isolated by excluding other cell types, such as cardiomyocytes, vascular smooth muscle cells (VSMCs) and endothelial cells (ECs), and hematopoietic cells. Additionally, some markers, such as discoidin domain-containing receptor 2 (DDR2), fibroblast-specific protein 1 (FSP1), stem-cell antigen 1 (Sca1), and vimentin, were initially used to identify CFs; however, these were not unique markers of fibroblasts and were also not expressed by the whole CF population [6,7,8,9]. Currently, other proteins are extensively used to identify the CF population: platelet-derived growth factor receptor alpha (PDGFRα), collagen1α1 (Col1a1), transcription factor 21 (Tcf21), and CD90 (*Thy1* gene), although the latter only comprises around 60–70% of the total CF population [8,10,11,12]. MEFSK4 antibodies have also been widely used to identify CFs, as they co-stain with PDGFRα and Col1a1 markers [9]. On the other hand, α-Smooth muscle actin (SMA) and Periostin (Postn) are markers for myofibroblasts [11,12], but do not label fibroblasts in the absence of myofibroblast conversion. Moreover, α-SMA lacks specificity for the fibroblast lineage, as it is also highly expressed in VSMCs. Nowadays, the most reliable markers for fibroblasts in the myocardium are the transcription factor (TF) Tcf21 [13,14] and the growth factor receptor PDGFRα [15]. In mice, the PDGFRα-Green fluorescent protein (GFP) reporter line was found to specifically label interstitial and perivascular fibroblasts without any overlap with vascular mural cells [16]. However, activated fibroblasts after cardiac injury exhibit a reduced expression of Tcf21 and PDGFRα [8,10,11,17,18]. This remarkable phenotypic plasticity exhibited by CFs under conditions of stress adds additional challenges to the identification of fibroblasts using a single marker. Thus, the reliable characterization of CFs may require the combined use of fibroblast-related markers (Table S1) along with exclusion criteria for other cell types [6,8,9,14,19,20,21,22,23,24,25,26,27,28,29,30,31,32,33,34,35,36,37,38,39].

The use of reporter mouse models developed under fibroblast-specific promoters has allowed the identification of different CF states that emerge during the adverse remodeling process that eventually leads to cardiac fibrosis [8,10]. Several studies have outlined that quiescent CFs stimulated after cardiac injury acquire a pro-inflammatory (cytokine secretion) phenotype that contributes to the immune response and quickly re-enter the cell cycle and begin to proliferate. Later, they differentiate into myofibroblasts to finally lead to a specific cell state, characterized by the expression of a tendon-like gene program, known as matrifibrocyte. All these findings highlight the phenotypic plasticity and functional diversity that exist behind the conventional pro-fibrotic role of fibroblast to myofibroblast conversion.

CFs can be activated via numerous signaling pathways following cardiac injury, with canonical TGF-β being the master pathway that orchestrates fibrosis across different tissues. Both the de novo synthesis of TGF-β produced by immune cells and the activation of TGF-β from latent myocardial stores contribute to a marked increase in bioactive TGF-β after cardiac injury. Active TGFβ binds the heterodimeric kinase receptor TGFβR1/2 located on the cell membrane, leading to the phosphorylation of SMAD2 and SMAD3 TF. Once phosphorylated, these TFs form a complex with SMAD4 and translocate into the nucleus to drive a pro-fibrotic transcriptional response characterized by a high expression of collagens, contractile proteins, and structural and enzymatic ECM components that mediate adverse tissue remodeling [40]. In addition to this canonical pathway, other pathways, like non-canonical TGF-β (via p38/Mitogen-Activated Protein Kinases (MAPK) [41] or Protein kinase B/Phosphoinositide 3-kinase (Akt/PI3K) [42]), or the activation of mechanotransduction (via TEA domain family member 1-yes-associated protein 1 (Tead-YAP1)/Transcriptional co-activator with PDZ-binding motif (TAZ)) pathways [43] also trigger myofibroblast conversion and cardiac fibrosis in distinct CVDs.

As a result, many experimental anti-fibrotic therapies have been designed against different targets implicated in these pro-fibrotic signaling pathways. For example, the pharmacological inhibition of TGFβR1 in ex vivo cultured myofibroblasts derived from heart failure patients was able to dedifferentiate them into a less activated state with a decreased expression of pro-fibrotic genes, suggesting its potential as a therapeutic reagent [44]. Additionally, a clinical evaluation of the Food and Drug Administration (FDA)’s approved drug pirfenidone, which inhibits the production of TGF-β cytokine, has recently shown encouraging results for ameliorating cardiac fibrosis in human heart failure patients [45]. Furthermore, the fibroblast-specific deletion of YAP has allowed the abrogation of cardiac fibrosis in animal models [46,47], confirming that targeting mechanically sensitive pathways might also be a potential therapeutic approach.

On the other hand, the maintenance of CF quiescence under homeostatic conditions is also regulated by distinct molecular pathways, whose perturbations are involved in the pathogenesis of cardiac fibrosis. For instance, the Hippo pathway has been shown to restrain fibroblast activation in healthy adult hearts, acting as a negative regulator of fibrotic phenotypes [43]. Moreover, the transcription factor SMAD7 acts as a repressor of the TGF-β signaling pathway, preventing CF activation in uninjured hearts [48].

## 3. Single-Cell Transcriptomics in Cardiac Fibrosis

Omics technologies have emerged as powerful tools in the understanding of CVDs. Specially, single-cell transcriptomic studies have uncovered a complex regulation of fibrosis identifying novel fibroblast subpopulations that appear in the adult heart after cardiac injury, as well as their evolution during the progression of the disease (Table 1).

The first single-cell RNA-sequencing (RNA-seq) studies were carried out on heart cells derived from healthy or fibrotic mouse models. Skelly et al. were one of the pioneer groups in identifying new fibroblast subpopulations by performing single-cell RNA-seq on cells isolated from healthy murine hearts [49]. Of note, they discovered a subpopulation of Col1a1-expressing CFs that lacked *Pdgfrα* and *Tcf21* but expressed Wnt pathway-related genes, such as *Dkk3*, *Wif1*, *Tbx20*, and *Frzb*. The presence of this fibroblast subpopulation was confirmed by Faberhi et al. in both healthy and infarcted hearts obtained from a Pdgfrα-GFP mouse model [10]. In this same study, they also defined other CF subpopulations, including activated and proliferating fibroblasts, which predominated at day 3 post-infarction, and multiple myofibroblast subgroups, which appeared since day 7 post-infarction and expressed different pro-fibrotic or anti-fibrotic transcriptomic profiles. Although this result suggested that fibrosis is self-limiting, the functional role of the distinct myofibroblast subsets has not been further investigated. In another study that integrated single-cell RNA-seq with Assay for Transposase-Accessible Chromatin sequencing (ATAC-seq) technology, a CD200^+^/CD146^−^ fibroblast reparative cluster that emerged after MI was identified. This specific fibroblast subpopulation was characterized by a reparative and pro-fibrotic transcriptional profile and displayed increased activity of SRY-Box Transcription Factor 9 (Sox9) [18]. In addition, Forte et al. detailed the presence of the different fibroblast subpopulations that sequentially emerge in the heart after MI in a Wt1-ZsGreen1 mouse model [50]. They defined three clusters of CFs prevalent under homeostatic conditions of the healthy heart, including homeostatic epicardium-derived, endocardial-derived, and progenitor-like fibroblasts. Homeostatic fibroblasts declined after cardiac injury, when suddenly appeared a transient subpopulation of injury-response fibroblasts, characterized by an inflammatory transcriptomic profile. From day 3 to 7 after MI-emerged myofibroblasts showed a downregulated expression of stem cell markers and an overexpression of ECM- and contractile-related genes. At later stages after infarction, they confirmed the presence of matrifibrocytes in the mature fibrotic scar, which expressed genes more related to osteoblasts and chondrocytes. Another single-cell RNA-seq study was carried out to identify CF subpopulations that arise in a mouse model of cardiac fibrosis induced by hypertension generated via chronic angiotensin-II administration [51]. This analysis confirmed the presence of the Wingless-related integration site (Wnt) signaling-related subpopulation, previously found in the infarcted myocardium, and identified two pro-fibrotic fibroblast clusters, characterized by the expression of *Cilp* and *Thbs4* markers. However, no α-SMA expressing myofibroblasts were detected, suggesting that this fibroblast state is not required in all CVDs and that different injury models of cardiac fibrosis may exhibit heterogeneous subpopulations of CFs. Furthermore, Peisker et al. performed a single-cell study in hearts derived from a mouse model of cardiac fibrosis induced by pressure overload, through a transverse aortic constriction (TAC) surgery, and described that the pro-fibrotic transformation of CFs was mainly governed by Tead1 TF [52].

In recent years, single-cell RNA-seq analyses of CF subpopulations have also been conducted in the human heart. First, Koenig et al. obtained 220,752 nuclei and 49,723 cells from the hearts of a large cohort of 28 non-diseased donors and 18 donors with non-ischemic dilated cardiomyopathy. As expected, they found a decrease in resting fibroblast populations, whereas an increase in activated fibroblast subsets in diseased patients in contrast to donor hearts [53]. Moreover, they associated these phenotypic shifts with the dysregulation of the pro-fibrotic transcription factor MEOX1. More recently, the largest single-nucleus RNA-seq dataset of human cardiomyopathy (including dilatative, arrhythmogenic, and hypertrophic heart diseases) was published. This dataset allowed the identification of a unique subset of activated fibroblasts that was almost entirely absent in non-failing hearts, being a novel potential population for the development of targeted anti-fibrotic therapies [54]. Moreover, this population of activated CFs showed an increased expression of known fibrotic markers like POSTN and COL1A1, whereas the resting PDGFRα marker was downregulated, in line with previous mouse studies. Furthermore, by integrating spatial transcriptomics, single-nucleus RNA-seq, and single-nucleus ATAC-seq, Kuppe et al. reported a comprehensive map delineating the enrichment of distinct CF subpopulations in different zones of the human infarcted heart. This study led to the identification of a differentiation trajectory from SCARA5^+^ PCOLCE2^+^ fibroblasts to POSTN+ ECM-producing myofibroblasts, which seemed to be regulated by several pro-fibrotic transcription factors, including MEOX1 and TEADs [55]. Finally, it has been recently published a preprint paper dissecting the immune-fibrosis axis in human CVDs by combining cellular indexing of transcriptome and epitope sequencing (CITE-seq), single-nucleus RNA-seq, and spatial transcriptomics in human hearts from non-diseased donors and ischemic and non-ischemic chronic cardiomyopathy patients [56]. The authors investigated CF subpopulations in chronic heart failure patients and identified a pro-fibrotic fibroblast trajectory, marked by fibroblast activation protein alpha (FAP) and POSTN expression that was modulated by macrophages and interleukin 1beta (IL1β) inflammatory cytokine and highly contributed to cardiac fibrosis. These findings suggest the broader therapeutic potential of targeting IL1β cytokine as a promising anti-fibrotic therapy.

Single-cell RNA-seq studies not only have allowed the delineation of fibroblast heterogeneity on the fibrotic myocardium but also the discovery of novel markers for the identification of activated CFs, such as cytoskeleton-associated protein4 (Ckap4) [57], collagen triple helix repeat-containing 1 (Cthrc1) [18], and hydroxysteroid 11-beta dehydrogenase 1 (Hsd11b1) [58]. Furthermore, a recently published broader cross-tissue comparison of mouse single-cell RNA-seq data identified markers of universal fibroblasts, such as peptidase inhibitor 16 (Pi16) and collagen type XV alpha 1 chain (Col15a1) [59]. They claimed that universal fibroblast subpopulations, characterized by the expression of these markers, could be considered “progenitor fibroblasts” as they exist across tissues and lead to specialized fibroblasts within each tissue in homeostatic conditions and to activated fibroblasts in diseased conditions.

The era of omics technologies has yielded numerous advances in the delineation of fibroblast heterogeneity and phenotype in different disease settings, which could facilitate the design of rational, highly targeted anti-fibrotic therapies, ultimately allowing the specific inhibition, ablation, or reprogramming of pathological fibroblast subpopulations while preserving essential homeostatic fibroblasts’ function.

## 4. Epigenetics in Cardiac Fibrosis

**Figure 1:**
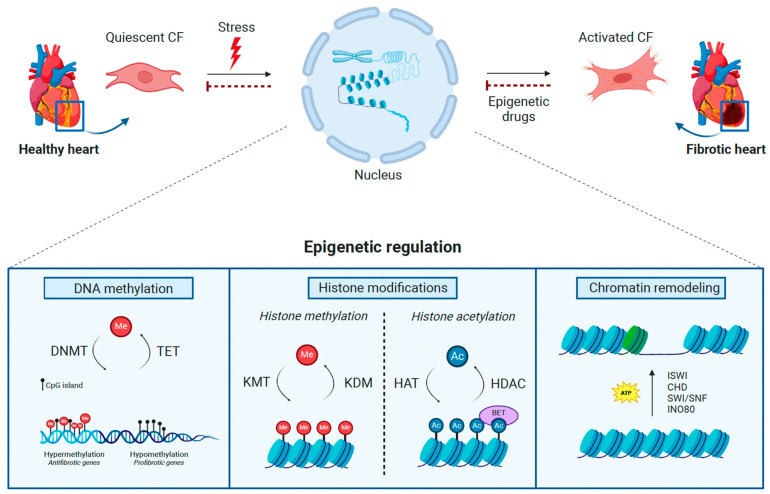
Epigenetic regulation in cardiac fibrosis. In response to pathological stresses, the epigenetic machinery is activated to promote cardiac fibroblast (CF) activation, leading to cardiac fibrosis. The complex interaction between DNA methyltransferases (DNMT), tet methylcytosine dioxygenases (TET) (DNA methylation), lysine methyltransferases (KMT), lysine demethylases (KDM), histone deacetylases (HDAC), histone acetyltransferases (HAT), bromodomain and extra-terminal motif proteins (BET) (Histone modifications)**,** and ISWI, CHD, SWI/SNF, and INO80 complexes (Chromatin remodeling) govern the methylation (Me) of DNA and histones, the acetylation (Ac) of histones, and changes in nucleosomes, consequently impacting the progression of cardiac fibrosis. The therapeutic administration of epigenetic drugs targeting these epigenetic regulators may revert the pathological consequences of specific epigenetic mechanisms in the development of fibrotic disease. *This figure was created with BioRender.com*.

In recent years, more and more studies have shown how epigenetic mechanisms may play an essential role in the development and progression of CVDs. Through these processes, gene expression is regulated independently of changes in DNA sequence acting through DNA methylation, histone modifications, and nucleosome remodeling (Figure 1). These modifications modulate chromatin structures and, therefore, the binding of transcription factors to control gene expression. Because of their immediacy, epigenetic mechanisms are the main cellular processes regulating responses to environmental changes, as they occur after cardiac injury, and their perturbation has been directly linked to alterations in cellular and organ functions at the onset of not only cardiac fibrosis but many other diseases, such as cancer. Furthermore, they are also reversible, representing a promising field for the development of therapeutic strategies. Hence, a growing number of studies are focusing on the investigation of epigenetic changes in CVDs for the development of novel anti-fibrotic approaches. For instance, DNA methylation has been found to be increased in fibrotic hearts derived from human patients and the acetylation of histones seems to be a relevant mechanism regulating cardiac fibrosis, allowing the development of anti-fibrotic drugs through the reversion of these epigenetic marks.

### 4.1. DNA Methylation

In the heart, DNA methylation has been shown to play a critical role in the control of gene expression and its aberrant regulation has been linked to the occurrence of CVDs [60]. DNA methylation is associated with transcriptional silencing. This epigenetic mark is written and erased by specific epigenetic factors known, respectively, as DNA methyltransferases (DNMT) and tet methylcytosine dioxygenases (TET). DNMT add methyl groups to the cytosines of cytosine-phosphodiester bond-guanine (CpG) dinucleotides. Regions of repetitive CpG sites, known as CpG islands, are often found in gene promoters and, when methylated, silence gene expression by preventing transcription factors from DNA binding. Specifically, DNMT3a and DNMT3b catalyze de novo DNA methylation, a pattern that is maintained by DNMT1 during DNA replication [61,62]. Conversely, TET enzymes directly oppose the activity of DNMT by erasing DNA methylation and, therefore, promote gene expression [63].

In the failing human heart, fibrosis has been correlated with enhanced DNA methylation [64]. Particularly, CFs exhibit increased DNA methylation status under the ischemic conditions that trigger cardiac fibrosis, which has been associated with an increased expression of DNMT1, DNMT3a, and DNMT3b. The DNMT1-mediated methylation of candidate genes has been shown to promote CF activation and proliferation [65,66]. Furthermore, it has been described that DNMT3a promotes fibroblast activation via the extracellular signal-regulated kinase 1/2 (ERK1/2) signaling pathway in a rat model of cardiac fibrosis and its inhibition has been shown to mitigate the fibrotic disease [67,68]. Like DNMT3a, DNMT3b also promotes CF activation and fibrosis through the aberrant methylation of fibrotic-related genes, such as Ras protein activator like-1 (Rasal1) and Ras-association domain family 1 (Rassf1) [69]. During myocardial hypoxia, hypoxia-inducible factor (HIF)-1α upregulates DNMT1 and DNMT3b expression, increasing the methylation and synthesis of related fibrosis markers, like α-SMA [70]. The functional inhibition of DNMT proteins with 5-aza-2′-deoxycytidine has been shown to impair the upregulation of the α-SMA pro-fibrotic gene under hypoxic conditions [71] and reduce myocardial fibrosis in a hypertensive rat model [72]. In addition, the pharmacological inhibition of DNA methylation with RG-108 has been shown to prevent cardiac fibrosis in a pressure overload (TAC) murine model [73].

As described above, DNMT-related drugs possess a great potential for attenuating CF activation and fibrotic disease. Nevertheless, different DNMT factors have shown to display different mechanisms in different models of cardiac fibrosis, making it difficult to develop a precise therapy without off-target effects. Because of that, the use of DNA methylation inhibitors for the treatment of cardiac fibrosis is still in preclinical stages, despite their promising potential. Further studies investigating DNA methylation will enable the application of targeted epigenetic therapies to the clinic for diagnosing and treating cardiac fibrosis [74].

### 4.2. Histone Modifications

Histone (H) modifications constitute an important epigenetic mechanism regulating fibrotic gene expression, and the fibrogenic role of histone modifiers has been determined at the onset of many CVDs. Histones form the core structure of chromatin, called the nucleosome, in which 147 base pairs of DNA are wrapped around a histone octamer comprising an H3–H4 tetramer flanked by two H2A–H2B dimers [75]. Arrays of nucleosomes fold and compact into more complex higher-order structures that, ultimately, form chromosomes [76]. Linker histone H1 binds to the DNA entry and exit site of a nucleosome and to linker DNA in order to promote intra-nucleosome interactions and chromatin compaction [77]. Histones pack DNA into chromatin being crucial in epigenetic regulation by altering the accessibility of DNA through histone post-translational modifications. Histone modifications, including methylation, acetylation, phosphorylation, and monoubiquitination, are performed at amino acids near the N-terminal tail of histone proteins. Among them, methylation and acetylation have been the most deeply studied in the pathogenesis of cardiac fibrosis and will be described next.

#### 4.2.1. Histone Methylation

Histone methylation can be associated with either active or repressed gene transcription depending on the amino acid modified, the state of methylation (mono-, di-, or tri-methylation) and its genomic distribution (promoters, enhancers, or gene bodies). For instance, H3K4me1/2/3, H3K9me1, H3K27me1, H3K36me1/2/3, and H3k79me1/2/3 are active marks associated with gene activation, whereas H3K9me3, H3K27me3, and H4K20me2/3 are repressive marks associated with gene silencing. Histone methylation is catalyzed by histone methyltransferases (HMTs), which can be divided into protein lysine methyltransferases (KMTs) and protein arginine methyltransferases (PRMTs), and removed by histone lysine demethylase (KDM), which are broadly divided into two families, lysine specific demethylases (LSDs) and JMJC domain-containing family (JMJD).

It has been described that both KMT and KDM play important roles in the pathogenesis of CVDs. For instance, the H3K4me3 epigenetic mark has been found upregulated at the promoter region of the Smad3 pro-fibrotic gene after the TGFβ stimulation of CFs [78]. Also, the specific deletion of the SET1 protein complex, which catalyzes the di- and tri-methylation of H3K4 at the promoters of actively transcribed genes, has been shown to reduce cardiac fibrosis in a mouse model of hypertension caused by angiotensin-II administration [79]. Controversial pro- and anti-fibrotic roles have been described for EZH2 KMT, which writes the H3K27me3-repressive mark. On the one hand, a reduced expression of EZH2, and therefore of H3K27me3 levels, has been associated with the induction of pro-fibrotic genes in the skin and the heart [80,81,82]. On the other hand, a pro-fibrotic role for EZH2 has been described in liver, lung, and heart fibrotic diseases [83,84,85,86]. In addition, the demethylation of the histone mark H3K9me2 governed by KDM3a has been shown to cause fibrosis in a mouse model of pressure overload. This pro-fibrotic effect was counteracted by the administration of the pan-KDM inhibitor JIB-04 [87].

#### 4.2.2. Histone Acetylation

On the other hand, histone acetylation is associated with active gene transcription as the acetyl group adds a negative charge, which repels negatively charged DNA, resulting in a relaxed chromatin structure that allows transcription factor binding to initiate gene expression [88]. Histone acetylation is a highly dynamic process regulated by two competitive enzymatic families: histone acetyltransferases (HATs) and histone deacetylases (HDACs), which, respectively, add and remove acetyl groups. Moreover, it is known that histone acetylation marks are read by the bromodomain and extra-terminal motif (BET) family of epigenetic factors, which act as reader proteins and, through the binding to acetylated lysine residues of histone tails, recruit other regulatory factors to activate transcription. All these epigenetic families involved in histone acetylation have been shown to play key roles in the development of fibrotic disease.

Firstly, the HAT family is further subclassified into three categories (GNAT, MYST, and CBP/P300) depending on substrate-interacting preferences. For instance, the GNAT family acetylates histone H3, while the MYST family has a higher affinity for histone H4 [89,90,91]. The CBP/P300 family has shown both H3 and H4 substrate preferences, as well as some affinity for non-histone proteins, such as transcription factors or other coregulators [92,93]. The role of CBP/P300 in cardiac fibrosis has been widely described, acting as a coactivator of the TGF-β signaling pathway/Smad3 transcription factor and epigenetically regulating the expression of collagen pro-fibrotic genes [94,95,96]. Furthermore, P300 inhibitors, like curcumin, have been shown to reduce cardiac fibrosis in different animal models [97,98,99]. However, these inhibitors are scarcely selective and widely inhibit all HAT factors; so, further studies are needed to faithfully assess the efficacy of specific CBP/P300 inhibition in the treatment of fibrotic diseases. More recently, the pro-fibrotic role of KAT8, a HAT belonging to the MYST family, has been demonstrated in murine models of liver and lung fibrosis [100,101]. However, KAT8’s pro-fibrotic role in the cardiac tissue is still undetermined.

Secondly, the HDAC family is a group of 18 distinct enzymes divided into four subclasses: class I (includes HDAC1, HDAC2, HDAC3, and HDAC8), class II (HDAC4, HDAC5, HDAC6, HDAC7, HDAC9, and HDAC10), class III (SIRT1-7), and class IV (HDAC11). Classes I, II, and IV belong to the classical HDAC family that deacetylates via a zinc-dependent mechanism, while class III HDAC, also known as sirtuins (Sirt), are mainly dependent on the NAD^+^ cofactor. Many studies have focused on the analysis of classical HDAC inhibitors for the treatment of cardiac fibrosis. For instance, the use of trichostatin, a pan-HDAC inhibitor, or of HDAC class I-specific inhibitors, like mocetinostat and PD-106, have been shown to be effective against cardiac fibrosis in various animal models of MI and hypertension, ameliorating heart function [102,103,104]. The anti-fibrotic effect of these inhibitors was in part due to the suppression of CF proliferation and the prevention of myofibroblast expansion [103,104]. Valproic acid (VPA) is another interesting HDAC inhibitor as it markedly attenuated cardiac fibrosis in a hypertensive rat model by inhibiting HDAC2 and HDAC8 activities [105]. In this regard, VPA has been also shown to impair pericyte-to-myofibroblast transition in a rat model of pressure overload by inhibiting HDAC4 [106]. Contrary to the pro-fibrotic roles of classical HDAC, sirtuins seem to have anti-fibrotic functions in the heart. For instance, it has been shown that Sirt2 is protective against cardiac fibrosis, hypertrophy, and dysfunction [107]. Moreover, Sirt3 and Sirt6 ameliorated cardiac fibrosis in a mouse model of pressure overload by regulating Smad3 transcription factor activity [108,109]. Finally, Sirt1 overactivation has also shown cardioprotective effects in a murine model of pressure overload [110].

Paradoxically, despite performing antagonistic functions, both HAT and classical HDAC epigenetic factors seem to be pro-fibrotic regulators of cardiac fibrosis. This could be explained by a crosstalk between them, and the third family implicated in the histone acetylation process: BET readers. There is evidence demonstrating that HDAC inhibition prevents proper HAT function, creating spurious acetyl histone marks that results in an altered localization of BET epigenetic factors and, in the end, impairing CF activation [111,112]. In this regard, targeting histone acetylation by inhibiting BET proteins represents a novel and promising approach for the treatment of fibrotic diseases, including cardiac fibrosis [113,114]. Of note, the protective effects of BET inhibitors seem to be due to the epigenetic regulation of the Meox1 pro-fibrotic factor, which triggers CF reversion into a more quiescent (less fibrotic) phenotype.

### 4.3. Chromatin Remodeling

Chromatin remodeling is a process that uses the energy of ATP hydrolysis to slide, eject, deposit, or alter the composition of nucleosomes by the action of chromatin remodeling complexes. This process may regulate gene expression by changing nucleosome structure along the chromatin and, therefore, the accessibility to DNA of transcription factors and other regulatory factors [115]. Chromatin remodeling complexes include four different families: ISWI, CHD, SWI/SNF, and INO80 [63], but all share a conserved ATPase domain, which confers them their catalytic activity. These complexes show distinct functionalities. For instance, the ISWI and CHD subfamilies are implicated in nucleosome assembly and organization, especially after replication, helping in the assembly and spacing of nucleosomes into DNA [116]. The SWI/SNF family mediate chromatin accessibility through nucleosome sliding or eviction, at promoters or enhancers, regulating gene transcription [117]. Whereas assembly remodelers promote gene silencing through the formation of tightly packed nucleosome arrays, access remodelers usually induce gene expression by opening the chromatin and exposing transcription factor binding sites. There are three main known complexes belonging to the SWI/SNF family of chromatin remodelers: BAF, PBAF, and non-canonical BAF. Finally, the INO80 family conducts the replication-independent editing of nucleosomes through the replacement of a particular histone with another canonical or histone variant [118]. The addition of histone variants into nucleosomes may affect gene expression by regulating the recruitment or repression of transcription factors to the DNA or generating labile nucleosomes, which can easily be ejected from the chromatin.

Despite having critical roles during cardiac developmental stages [119], chromatin remodelers are not studied at the onset of CVDs. Interestingly, some studies have suggested their hypothetical function as pro-fibrotic mediators. For instance, the PBAF-specific subunit responsible for chromatin recognition, Pbrm1, is a key regulator of the TGFβ/BMP signaling pathway that triggers osteogenic differentiation in mesenchymal cells [120]. Furthermore, Smarca4, a specific subunit of the non-canonical BAF complex, has shown controversial fibrotic roles, respectively, promoting and ameliorating liver and lung fibrotic diseases [121,122]. However, further studies are needed to better understand the roles and mechanisms of chromatin remodeling complexes in the development of fibrosis.

Even though classical epigenetic modifications, such as methylation and acetylation, have an undisputable role in the pathogenesis of cardiac fibrosis, the specific function of the targeted epigenetic factors that govern these processes remains to be cleared, and further studies are needed that will determine the role of chromatin remodeling complexes.

## 5. Conclusions and Future Directions

In this review, we have briefly summarized the advances that have been made in recent years in understanding the pathogenesis of cardiac fibrosis, with a focus on the insights gained by the transcriptomic analyses and the relevance of epigenetic regulation in anti-fibrotic therapy.

The development of multi-omics technologies has increased our understanding of the cellular and molecular mechanisms that trigger CF activation and cardiac fibrosis. Single-cell RNA-seq technology has allowed us to deconvolute the healthy and fibrotic hearts at a deep resolution, discovering an unexpected cellular heterogeneity. To date, this technology is evolving and providing molecular information not only within a single cell but also across different cells with high spatial and temporal accuracy. The addition of spatiotemporal context to these datasets will be critical for understanding changes in the fibrotic niche and pathological cellular crosstalks that occur after cardiac disease [123]. However, spatial transcriptomics are currently limited, as they can only target a low number of genes, which have to be previously selected in a panel, and are usually biased towards highly abundant transcripts of the more expressed genes. Not only deep or resolution needs to be improved, but also a cooperative effort is needed to standardize a common annotation of cell types and states found in healthy and fibrotic hearts, which will facilitate a more precise comparison between studies and the procurance of more reliable conclusions in the interpretation of the derived data. Finally, it is important to mention that these technologies face an inherent obstacle in their use as diagnostic tools to assess cardiac fibrosis, as they require the acquisition of fresh heart tissue that is a highly invasive process not feasible in live patients. Nevertheless, these technologies accelerate our understanding of adverse cardiac remodeling and contribute to the discovery of new potential therapeutic targets for the treatment of cardiac fibrosis.

As such, the further understanding of epigenetic regulation has led to the discovery of small-molecule inhibitors as promising therapeutic options in various diseases, like cancer. In recent years, numerous studies, highlighted in this review, have revealed the essential role of epigenetics in the pathogenesis of cardiac fibrosis and the anti-fibrotic effect of many inhibitors, like those targeted against HDAC or BET proteins. However, none of them have been evaluated for fibrotic disease in clinical trials due to two important limitations still to be resolved: the lack of precise knowledge about specific epigenetic mechanisms regulating cardiac fibrosis and the possible adverse effects of pan-inhibitors in other tissues or cells.

Importantly, epigenetic therapy has emerged as a promising field because of the reversible nature of epigenetic modifications. However, the preclinical research of epigenetics in fibrotic diseases is relatively immature compared with other pathologies. Different results have been found for the same epigenetic drug in different studies depending on the fibrotic animal model, the fibrotic stages, or the dosage used. Therefore, further studies are needed to precisely evaluate the therapeutic effect of epigenetic drugs and to precisely understand the anti-fibrotic mechanisms regulated after their administration. These studies will facilitate the development of more specific inhibitors that exclusively target the catalytic activity of the epigenetic factor (as opposed to pan-inhibitors) and probably allow a more successful application of the epigenetic therapy into the clinic to treat the fibrotic disease.

Another major challenge of epigenetic therapies is the lack of cell specificity, which may result in deleterious effects for other organs and cells. The evolution of cell-specific delivery systems will provide solutions with higher specificity. Studies focused on selectively targeting activated CFs for drug delivery are of great interest for anti-fibrotic therapy development, as they are the main effector cells in the progression of the disease. These include viral vectors or functionalized nanoparticles coated with fibroblast antigens [124]. For instance, it has been shown the effectiveness of specifically target activated CFs in a mouse model of MI with the systemic administration of a cardiotropic adeno-associated viral vector (serotype 9), which carried a Postn promoter [125]. More recently, it has been demonstrated that engineered T cells, which expressed a chimeric antigen receptor against FAP protein, were able to selectively target pathogenic CFs in a mouse model of MI [126,127]. Fortunately, single-cell RNA-seq techniques have allowed the identification of heterogenous fibroblast subpopulations, identifying distinct functional states that favor the development of targeted therapies against the most fibrotic clusters minimizing off-target effects.

In conclusion, the application of the latest advances of omics technologies joined with a deeper understanding of epigenetic mechanisms that trigger the fibrotic response should facilitate a more precise delineation of the fibrotic disease in the heart as well as the design of rational, highly targeted anti-fibrotic therapies.

## Figures and Tables

**Table 1:** Single-cell transcriptomic datasets exploring cardiac fibrosis.

Title	Assay	Species	Tissue	Cell Types	Cell No.	Main Finding	Ref.
Single-Cell transcriptional profiling reveals cellular diversity and intercommunication in the mouse heart	Single-cell RNA-seq	Mouse	Healthy heart	Non-myocyte cells	10,519	This study explores cardiac cellular diversity and provides unique insights into the structure and function of the cardiac cellulome.	[49]
Single-cell expression profiling reveals dynamic flux of cardiac stromal, vascular, and immune cells in health and injury	Single-cell RNA-seq	Mouse	Healthy and MI hearts	Non-myocyte cells and enriched (Pdgfra-GFP+) fibroblast lineage cells	16,787	This study describes fibroblast lineage trajectory in both sham and MI hearts.	[10]
Single-cell RNA sequencing analysis reveals a crucial role for CTHRC1 (collagen triple helix repeat containing 1) cardiac fibroblasts after myocardial infarction	Single-cell RNA-seq	Mouse	Healthy and MI hearts	Non-myocyte cells and enriched (Col1a1-GFP+) fibroblast lineage cells	29,176	This study identifies a subpopulation reparative CFs characterized by a distinct transcriptional profile, including Cthrc1.	[18]
Dynamic interstitial cell response during myocardial infarction predicts resilience to rupture in genetically diverse mice	Single-cell RNA-seq	Mouse	Healthy and MI hearts	Non-myocyte cells and enriched (Wt1-GFP+) epicardial-derived cells	36,847	This study identifies multiple epicardial-derived fibroblast subtypes whose sequential appearance defined post-MI phases.	[50]
High-resolution transcriptomic profiling of the heart during chronic stress reveals cellular drivers of cardiac fibrosis and hypertrophy	Single-cell RNA-seq	Mouse	Healthy and chronic-injured hearts	Cardiomyocytes and non-myocyte cells	7474	This study maps the cardiac cellular landscape uncovering two fibrotic fibroblast populations, Fibroblast-Cilp and Fibroblast-Thbs4.	[51]
Mapping the cardiac vascular niche in heart failure	Single-cell RNA-seq	Mouse	Healthy and chronic-injured hearts	Non-myocyte cells	77,602	This study characterizes a specific fibroblast subpopulation that acquires Thbs4 and Tead1 expression and expands after injury, driving cardiac fibrosis.	[52]
Single-cell transcriptomics reveals cell-type specific diversification in human heart failure	Single-cell and single-nucleus RNA-seq	Human	Healthy and chronic-injured left ventricle hearts	Cardiomyocytes and non-myocyte cells	49,723 cells/220,752 nuclei	This study identifies cell type-specific transcriptional programs and disease-associated cell states that emerge in the context of heart failure.	[53]
Single-nucleus profiling of human dilated and hypertrophic cardiomyopathy	Single-nucleus RNA-seq	Human	Healthy and chronic-injured hearts	Cardiomyocytes and non-myocyte cells	592,689 nuclei	This study defines molecular alterations in the failing heart and identifies a unique population of activated fibroblasts exclusively found in injured hearts.	[54]
Spatial multiomic map of human myocardial infarction	Single-nucleus RNA-seq, single-nucleus ATAC-seq and spatial sequencing	Human	Healthy and MI hearts	Cardiomyocytes and non-myocyte cells	191,795	This study uses spatial multiomics to identify important cell niches, cell states, and cell interactions in the infarcted cardiac tissue.	[55]
Targeting immune-Fibroblast Crosstalk in Myocardial Infarction and Cardiac Fibrosis	CITE-seq, single-nucleus RNA-seq, single-nucleus ATAC-seq and spatial sequencing	Human	Healthy and chronic-injured left ventricles hearts	Non-myocyte cells	143,804	This study characterizes the inflammatory–fibrosis axis in the human-infarcted heart describing a macrophage–fibroblast crosstalk driven by IL-1β that promotes CF activation.	[56]
Single-cell sequencing of the healthy and diseased Heart reveals cytoskeleton-associated protein 4 as a new modulator of fibroblasts activation	Single-cell RNA-seq	Mouse	Healthy and MI hearts	Cardiomyocytes and non-myocyte cells	935	This study identifies disease-specific subpopulations of various cell types in the heart, highlighting Ckap4 as a specific marker of activated CFs.	[57]
Single-cell dual-omics reveals the transcriptomic and epigenomic diversity of cardiac non-myocytes	Single-cell RNA-seq and single-cell ATAC-seq	Mouse	Healthy heart	Non-myocyte cells	12,779	This study characterizes the transcriptome and epigenome of non-myocyte cells of the heart discovering CF subpopulations with unique functional states.	[58]
Cross-tissue organization of the fibroblast lineage	Single-cell RNA-seq	Mouse	17 tissues and 11 disease states (among them healthy and injured hearts)	Fibroblasts	230,000	This study describes the presence of universal Pi16+ and Col15a1+ fibroblasts that lead to specialized fibroblasts across steady-state tissues and to activated fibroblasts in disease conditions.	[59]

## Supplementary Materials

The following supporting information can be downloaded at: https://www.mdpi.com/article/10.3390/ijms25116004/s1.

## Abbreviations

Akt/PI3K	Protein kinase B/Phosphoinositide 3-kinase
ATAC-seq	Assay for Transposase-Accessible Chromatin sequencing
BET	Bromodomain and extra-terminal motif
CITE-seq	Cellular indexing of transcriptome and epitope sequencing
CF	Cardiac fibroblasts
Ckap4	Cytoskeleton-associated protein4
Col1a1	Collagen1α1
Col15a1	Collagen type XV alpha 1 chain
Cthrc1	Collagen triple helix repeat-containing 1
CpG	Cytosine-phosphodiester bond-guanine
CVD	Cardiovascular diseases
DDR2	Discoidin domain-containing receptor 2
DNMT	DNA methyltransferases
ECs	Endothelial cells
ECM	Extracellular matrix
ERK1/2	Extracellular signal-regulated kinase 1/2
FAP	Fibroblast activation protein alpha
FDA	Food and Drug Administration
GFP	Green fluorescent protein
FSP1	Fibroblast-specific protein 1
H	Histone
HAT	Histone acetylases
HDAC	Histone deacetylases
HIF	Hypoxia-inducible factor
HMT	Histone methyltransferases
HSD11B1	Hydroxysteroid 11-beta dehydrogenase 1
IL1β	Interleukin 1beta
JMJD	JMJC domain-containing family
KDM	Histone lysine demethylase
KMT	Lysine methyltransferases
LSD	Lysine specific demethylases
MAPK	Mitogen-activated protein kinases
MI	Myocardial infarction
Pi16	Peptidase inhibitor 16
PDGFRα	Platelet-derived growth factor receptor alpha
PRMT	Arginine methyltransferases
Rasal1	Ras protein activator like-1 (Rasal1)
Rassf1	Ras-association domain family 1
RNA-seq	RNA sequencing
SMA	Smooth muscle actin
SIRT	Sirtuins
Sox9	SRY-Box Transcription Factor 9
Sca1	stem-cell antigen 1
TAZ	Transcriptional co-activator with PDZ-binding motif
Tead-YAP1	TEA domain family member 1-yes-associated protein 1
TET	Tet methylcytosine dioxygenases
TF	Transcription factor
Tcf21	Transcription factor 21
TAC	Transverse aortic constriction
VPA	Valproic acid
VSMCs	Vascular smooth muscle cells
Wnt	Wingless-related integration site
