# Supplementary material for: Fibroblast Diversity and Epigenetic Regulation in Cardiac Fibrosis
Source: Int J Mol Sci. 2024 May 30;25(11):6004. doi: 10.3390/ijms25116004 (PMC11172550; doi:10.3390/ijms25116004)
Supplement: Supplementary file 1 [file ijms-25-06004-s001.zip › ijms-3022438-supplementary.pdf]

Table S1. Sensitivity and specificity of markers used to identify CF. Adapted from Humeres C. & Frangogiannis G. JACC: Basic to Translational Science. 2019 [19]

Marker	Sensitivity	Specificity	Reference
**DDR2**	Labels all resting CF and subpopulations of activated CF	Also expressed by VSMC, EC	[20–23]
**FSP1**	Label a subpopulation of CF (resting and activated)	Also express by immune cells, VSMC, EC	[6,24,25]
**SCA1**	Label a subpopulation of CF (resting and activated)	Also expressed by pericytes, EC	[9,26]
**VIMENTIN**	Labels all CF (resting and activated)	Also expressed by pericytes, VSMC, EC	[27–29]
**PDGFRα**	Labels all CF (resting and activated)	Also expressed by some activated VSMC	[8,30,31]
**COL1A1**	Labels all CF (resting and activated)	Also expressed by pericytes, VSMC, EC	[30,32]
**TCF21**	Labels all CF (resting and activated)	Relatively specific for fibroblasts	[14,33]
**CD90**	Label a subpopulation of CF (resting and activated)	Also expressed by pericytes, immune cells, EC	[9,30,34]
**MEFSK4**	Labels all CF (resting and activated)	Also expressed by a small subpopulation of immune cells	[9]
**α-SMA**	Labels activated myofibroblasts	Also expressed by VSMC	[35,36]
**POSTN**	Labels neonatal and activated CF	Also expressed by some subpopulations of VSMC	[6,37–39]
